# Characterisation of *RHD* and *RHCE* variations in blood donors from Jazan Province, Southwestern Saudi Arabia

**DOI:** 10.1111/tme.70040

**Published:** 2025-10-28

**Authors:** Maymoon M. Madkhali, Mayisah Khormy, Abdullah A. Meshi, Bandar Kameli, Khaled Ghazwani, Ohoud Sufyani, Salha Hakami, Yahya Khawaji, Abdullah A. Mobarki, Khaled Essawi, Waleed Hakami, Yara Alyahyawi, Aymen M. Madkhali, Gasim Dobie, Hassan A. Hamali

**Affiliations:** ^1^ Regional Blood Bank Ministry of Health Jazan Saudi Arabia; ^2^ Blood Bank Department King Fahd Central Hospital, Ministry of Health Jazan Saudi Arabia; ^3^ Department of Medical Laboratory Technology, Faculty of Nursing and Health Sciences Jazan University Jazan Saudi Arabia

**Keywords:** allele, genotype, Jazan region, phenotype, Rh system, *RHD* and *RHCE*

## Abstract

**Background and Objectives:**

Rh is among the most important and highly polymorphic blood group systems due to the proximity of the *RHD* and *RHCE* genes, which encode numerous highly immunogenic antigens. However, in areas of Saudi Arabia with a high prevalence of hemoglobinopathy, the molecular characteristics of *RHD* and *RHCE* variations are lacking. This study focuses on characterizing the allelic and genotypic distributions of *RHD* and *RHCE* blood donors in Jazan, Saudi Arabia.

**Materials and Methods:**

All blood donors' records between June 2023 and December 2024 were reviewed. ID RHD XT was applied for 60 negative or weak RhD Saudi donors, while 354 Saudi and 110 non‐Saudi donors received *RHCE* genotyping using the ID CORE XT.

**Results:**

*RHD* deletion accounted for 76.7% of the RhD‐negative donors. RHCE*Ce (44.1%) was the most common *RHCE* allele in Saudis, followed by RHCE*ce (31.9%) and RHCE*cE (12.3%). Variant alleles including RHCEce*(733G), RHCEce*(733G,1006T), RHCE*ceAR, RHCE*ce(712G), and RHDr's‐RHCE*ce(733G,1006T) were detected in 11.72% in Saudi and 7.73% in non‐Saudi, while low‐ and high‐frequency antigens V, hrS, VS, and hrB were observed in Saudis with frequencies of 21.8%, 97.8%, 24.8%, and 93.2%, respectively. RHCE*ce/RHCE*Ce was the most prevalent genotype (26.8%). The non‐Saudi group displayed similar profiles, with RHCE*Ce (48.2%), RHCE*ce (30.9%), and RHCE*cE (13.2%). There were no significant differences between Saudi and non‐Saudi allele or genotype distributions using Fisher's exact test.

**Conclusion:**

The study represents the first molecular characterisation of *RHD* and *RHCE* alleles in Saudi Arabia, revealing marked allelic and genotypic diversity, with important implications for transfusion safety in the Jazan region, where hemoglobinopathies are prevalent.

## 1 INTRODUCTION

Following the ABO blood group system, the Rh system is clinically crucial and complex in transfusion medicine. The Rh system is highly polymorphic, comprising over 50 distinct antigens integrated into the red blood cell membrane, with the most significant antigens being D, C, c, E, and e.^1^, ^2^ The system's antigens are encoded by two genes (*RHD* and *RHCE*), of which *RHD* encodes different epitopes of the D antigen and *RHCE* encodes the C/c and E/e antigens.^3^, ^4^ These genes are closely linked on chromosome 1p36.11 and exhibit a high degree of homology. The *RHD* and *RHCE* genes vary geographically and among ethnicities and backgrounds. Over 400 *RHD* alleles and more than 150 *RHCE* alleles have been recognised.^5^ Gene conversion events between the two lead to the creation of hybrid genes, which can result in negative, weak, or partial phenotypes, such as red blood cell antigens.^1^, ^2^ These hybrid genes may produce altered proteins, resulting in a diverse array of Rh antigens, which could initiate immune responses and produce alloantibodies.^6^

Complexity in the Rh system arises from its extensive array of highly immunogenic antigens, which can trigger severe immunological reactions, such as alloimmunisation, resulting from Rh incompatibility and leading to life‐threatening conditions, such as haemolytic transfusion reactions and haemolytic disease of the foetus and newborn.^1^ The incidence of hemoglobinopathies such as sickle cell and thalassaemia in Saudi Arabia is high, particularly in the Eastern and Jazan regions.^7^, ^8^ Therefore, the rate of alloimmunisation and autoimmunisation among haemoglobinopathy patients remains a concern. Among these patients, the rate of alloimmunisation varies between regions, reaching up to 39%.^9^, ^10^, ^11^ In Jazan, the rate ranges from 7 to 13% with anti‐E and anti‐K being the most prevalent antibody specificities.^12^, ^13^ The genetic variations can lead to different Rh phenotypes, with significant implications for blood transfusion compatibility in patients receiving blood transfusions. Although serological tests are the gold standard for RhD detection in routine practices, they are sometimes ineffective and inaccurate.^14^ Thus, the identification and knowledge of these variants are clinically significant for finding suitably matched blood for transfusion‐dependent patients.^15^, ^16^

The frequency of blood group antigens varies among ethnicities. Extensive studies have been conducted to explore the distribution and frequency of Rh antigens globally. Studies have shown that the Rh‐positive phenotype is predominant in most populations, with frequencies ranging from 85% to 95%.^3^, ^4^, ^17^ The presence of a mixed ethnic population exhibits a disparity in red blood cell antigens, which may increase the risk of alloimmunisation.^18^ A 2022 report indicated that 29.7% (1148 out of 3863) of blood donors in Jazan were non‐Saudi and represented various ethnicities.^19^ Rh antigens are variable among blood donors in Saudi Arabia,^18^, ^20^, ^21^, ^22^, ^23^ including Jazan,^19^, ^24^, ^25^ due to their diverse ethnic backgrounds. The molecular background of the *RHD* and *RHCE* genes in Saudi Arabia is limited, especially in areas with a high prevalence of hemoglobinopathies, such as Jazan.^7^, ^8^ A recent study in Saudi Arabia revealed that most individuals with the RhD‐negative phenotype are likely to see a homozygous, complete deletion of the *RHD* gene and that *RHD* allele deletion is prevalent among the RhD‐positive phenotype.^26^ Furthermore, the *RHCE* gene has not been thoroughly studied in Saudi Arabia. Consequently, this study aims to investigate the variations in *RHD* and *RHCE* genes among Saudi blood donors in the Jazan region of southwestern Saudi Arabia.

## 2 MATERIALS AND METHODS

### 2.1 Data collection

This retrospective study was conducted between June 2023 and March 2024. Records of all blood donors recruited during this period were included for data acquisition. A total of 60 Saudi blood donors' records for RHD genotyping were reviewed. These individuals were initially serologically weak or RhD‐negative. A total of 354 Saudi blood donors' records and 110 non‐Saudi (mixed‐nationality) records for the *RHCE* genotype were reviewed. Blood bank donation registries, excluding donors with incomplete or duplicate data, were examined.

### 2.2 DNA extraction

Genomic DNA was extracted from EDTA‐anticoagulated whole blood samples using QIAamp DNA Blood Mini or FlexiGene kits (Qiagen, Hilden, Germany), following the kit instructions. The DNA concentration in the samples ranged from 8 to 80 ng/μL, with DNA purity (A260/A280 ratio) between 1.63 and 2.1, as measured by a Qubit 4 fluorometer (Thermo Fisher Scientific, Wilmington, DE, USA).

### 2.3 Genotyping

*RHD* genotyping was performed on individuals with weakly expressed and negative RhD phenotypes using *RHD* ID XT covering the alleles: RHD*weak D type 1, RHD*weak D type 2, RHD*weak D type 3, RHD*Pseudogene, RHD*DIIIa‐CE(3–7)‐D, and *RHD deletion*. *RHCE* genotyping was conducted on blood donors using ID CORE XT covering the following alleles: RHCE*CeCW, RHCE*CECW, RHCE*cE, RHCE*Ce, RHCE*CE, RHCE*ceAR, RHCE*ce(712G), RHCE*CeFV, RHCE*cEFM, RHCE*ce[733G], RHCE*ce(733G,1006T), RHCE*CeVG, RHCE*cE(712G,733G), RHCE*Ce(733G), RHD*r's‐RHCE*ce(733G,1006T), and RHCE*CE‐D[5, 7]‐CE. The targeted DNA regions are amplified through multiplex PCR using biotinylated dCTP.^27^ The PCR products obtained were subsequently hybridised onto oligonucleotide probes attached to microspheres and labelled with streptavidin‐conjugated phycoerythrin. The beads were then analysed using a Luminex® 200 system (Millipore Corporation, Billerica, MA, USA). The raw data were processed using ID CORE XT analysis software to obtain genotypes and predict blood group phenotypes.

## 3 ETHICAL CONSIDERATIONS

Ethical approval was obtained from the Institutional Review Board, Jazan, Ministry of Health, Saudi Arabia (no. 2402).

## 4 STATISTICAL ANALYSIS

Descriptive statistics were used to summarise *RHCE* allele and genotype frequencies, which are reported as counts and percentages. Fisher's exact test was employed to compare distributions between the Saudi and non‐Saudi donor groups. A *p*‐value of less than 0.05 was considered statistically significant. All analyses and visualisations were conducted using R software (2024.12.1 + 563).

## 5 RESULTS

### 5.1 *RHD* genotype frequencies among saudi blood donors

Most of the 60 Saudi blood donors who participated in this study (76.7%) showed full *RHD* gene deletion. Moreover, 18.3% of these individuals were categorised as ‘other than weak D types 1, 2 and 3’, and one donor exhibited weak D type 1 (1.7%). Furthermore, as shown in Table 1, two donors (3.3%) were heterozygous for the hybrid allele RHD*DIIIa‐CE (3–7)‐D.

**TABLE 1:** *RHD* allele frequencies among Saudi blood donors.

*RHD* genotype	ISBT allele name	Observations (*n*)	Frequency (%)
*RHD deletion*	RHD*01N.01	46	76.67
Other than weak D types 1, 2 and 3	^a^	11	18.33
*Weak D type 1*	RHD*01W.1	1	1.67
RHD*DIIIa‐CE(3‐7)‐D heterozygous	RHD*03N.01	2	3.33
Total		60	100

^a^ Other than weak D types 1, 2, and 3″ includes RHD variants not detected as weak D types 1, 2, or 3. The exact identification may require additional molecular analysis.

### 5.2 *RHCE* allele analysis

A total of 708 *RHCE* alleles from Saudi donors and 220 from non‐Saudi donors were analyzed. The most **common** alleles identified in the Saudi donors were RHCE*Ce 44.07% (312/708), RHCE*ce 31.92% (226/708), and RHCE*cE 12.29% (87/708). Individuals exhibited the RHCE*ce*(733G)* variant representing 9.46% (67/708) of the Saudi donors, while RHCE*ce(733G,1006T) was detected in 0.85% (6/708). RHCE*ceAR, RHCE*ce(712G), and RHDr's–RHCE*ce(733G,1006T) were detected in 0.71% (5/708), 0.42% (3/708), and 0.28% (2/708), respectively.

On the other hand, the non‐Saudi group displayed a largely similar profile in the detection of only four alleles: RHCE*Ce 48.18% (106/220), RHCE*ce 30.91% (68/220), and RHCE*cE 13.18% (29/220) being the dominant alleles, regardless of the rare variants that exist in Saudi Arabia. Fisher's exact test revealed that there was no statistically significant difference in the distribution of the *RHCE* allele between the Saudi and non‐Saudi donors (*p* = 0.5066). The comparative distribution is shown in Table 2.

**TABLE 2:** *RHCE* allele frequencies and observations among Saudi and non‐Saudi blood donors.

*RHCE* allele		Saudi donors		Non‐Saudi donors (mixed nationalities)	
*RHCE* allele	ISBT allele name	Observations (*n*)	Frequency (%)	Observations (*n*)	Frequency (%)
RHCE*Ce	RHCE*02	312	44.07	106	48.18
RHCE*ce	RHCE*01	226	31.92	68	30.91
RHCE*cE	RHCE*03	87	12.29	29	13.18
RHCE*ce(733G) ^a^	RHCE*01.20.01 RHCE*01.20.02	67	9.46	17	7.73
RHCE*ce(733G,1006T) ^a^	RHCE*01.20.03 RHCE*01.20.05	6	0.85	0	0.00
RHCE*ceAR	RHCE*01.04	5	0.71	0	0.00
RHCE*ce(712G) ^a^	RHCE*01.05 RHCE*01.08 RHCE*01.09	3	0.42	0	0.00
RHD*r's‐RHCE*ce(733G,1006T)	RHCE*01.20.03	2	0.28	0	0.00
Total		708	100	220	100

^a^ These alleles may have multiple possible ISBT designations depending on additional molecular changes. Exact identification may require further molecular investigation.

A total of 354 *RHCE* genotypes from Saudi donors and 110 from non‐Saudi donors were investigated. RHCE*ce/RHCE*Ce was the most common genotype in the Saudi donors, occurring in 26.84% (95), followed by RHCE*Ce/RHCE*Ce 19.49% (69), RHCE*Ce/RHCE*cE 13.28% (47), and RHCE*ce/RHCE*ce in 12.71% (45). Moreover, genotypes including the RHCE*ce variant alleles including RHCE*ce/RHCE*ce(733G) and RHCE*ce(733G)/RHCE*ce(733G) were found in 4.80% (17) and 2.26% (8), respectively. Less common and rare genotypes are shown in Table 3.

**TABLE 3:** *RHCE* genotype frequencies and their predicted phenotypes among Saudi and non‐Saudi donors.

		Saudi donors		Non‐Saudi donors (mixed nationalities)	
*RHCE* genotype	Predicted phenotype	Observations (*n*)	Frequency (%)	Observations (*n*)	Frequency (%)
RHCE*ce/RHCE*Ce	C+, E−, c+, e+, CW−, V−, hrS+, VS−, hrB+	95	26.84	31	28.18
RHCE*Ce/ RHCE*Ce	C+, E‐, c−, e+, CW−, V−, hrS+, VS−, hrB+	69	19.49	24	21.82
RHCE*Ce/RHCE*cE	C+, E+, c+, e+, CW−, V−, hrS+, VS−, hrB+	47	13.28	14	12.73
RHCE*ce/ RHCE*ce	C−, E−, c+, e+, CW−, V−, hrS+, VS−, hrB+	45	12.71	14	12.73
RHCE*Ce/ RHCE*ce(733G)	C+, partial c, e+, CW−, V+ hrS + VS + hrB+	28	7.91	12	10.91
RHCE*ce/ RHCE*cE	C−, E+, c+, e+, CW−, V+ hrS+, VS+, hrB+	24	6.78	7	6.36
RHCE*ce/RHCE*ce(733G)	C−, E−, c+, e+, CW−, V+, hrS+, VS+, hrB+	17	4.80	2	1.82
RHCE*ce(733G)/RHCE*ce(733G)	C‐, partial c, partial e, CW‐, V+ hrS + VS + hrB‐	8	2.26	0	0.00
RHCE*cE/ RHCE*cE	C−, E+, c+, e−, CW−, V−, hrS−, VS−, hrB−	4	1.13	3	2.73
RHCE*cE/ RHCE*ce(733G)	C−, E+, c+, partial e, CW−, V+, hrS+, VS+, hrB−	5	1.41	2	1.82
RHCE*ce/RHCE*ce(712G)	C−, E−, c+, e+, CW‐, V−, hrS+, VS−, hrB+	2	0.56	0	0.00
RHCE*ceAR/ RHCE*ceAR	C−, E−, partial c, partial e, CW−, V weak +, hrS−, VS−, hrB+	2	0.56	0	0.00
RHCE*ce(733G,1006T)/ RHCE*ce(733G,1006T)	C−, E−, partial c, partial e, CW−, V−, hrS+, VS+, hrB−	2	0.56	0	0.00
RHCE*Ce/RHCE*Ce(733G)	C+, E−, c−, e+, V, hrS, VS, and hrB are NA	0	0.00	1	0.91
RHCE*Ce/RHCE*ce(733G,1006T)	C+, partial c, e+, CW−, V−, hrS+, VS+, hrB−	1	0.28	0	0.00
RHCE*cE/RHCE*ce(712G)	C−, E + c + partial e, CW−, V−, hrS−, VS−, hrB+	1	0.28	0	0.00
RHCE*cE/RHCE*ce(733G,1006T)	C−, E+, c+, partial e, partial e, CW−, V−, hrS+, VS+, hrB−	1	0.28	0	0.00
RHCE*cE/RHD*r's‐RHCE*ce(733G,1006T)	Partial C, E+, partial c, partial e, CW−, V−, hrS+, VS+, hrB−	1	0.28	0	0.00
RHCE*ce/RHCE*ceAR	C−, E−, c+, e+, CW−, V weak +, hrS−, VS−, hrB+	1	0.28	0	0.00
RHCE*ce(733G)/RHD*r's‐RHCE*ce(733G,1006T)	Partial C, E−, partial c, partial e, CW−, V+, hrS+, VS+, hrB−	1	0.28	0	0.00
Total		354	100	110	100

Among the 110 non‐Saudi donors, the majority were from Yemen (73), followed by Egypt (16), Sudan (6), India (5), Pakistan (4), Syria (3), Jordan (1), Nepal (1), and the Philippines (1). The most frequently observed genotypes in the non‐Saudi donors were similar to those in the Saudi cohort. RHCE*ce/RHCE*Ce was detected in 28.18% (31), followed by RHCE*Ce/RHCE*Ce in 21.82% (21). RHCE*Ce/RHCE*cE and RHCE*ce/RHCE*ce were observed in 12.73% (14) for each genotype. Nonetheless, the frequency of variant genotypes involving RHCE*ce(733G) and other alleles was lower than that observed in Saudi donors. Fisher's exact test revealed no significant difference in *RHCE* genotype distribution between the Saudi and non‐Saudi donors (*p* = 0.8627). The *RHCE* genotype distribution for the two groups is presented in Table 3.

The most common Rh antigens found in the Saudi donors were e (98.84%) and c (80.48%), followed by C (68.36%) and E (23.44%). Low‐ and high‐frequency antigens, including V (21.8%), hrS (97.8%), VS (24.8%), and hrB (93.2%), were also identified; CW antigen (RH8) could not be found in the study cohort. Rh antigens typed by ID CORE XT from Saudi donors (this study) compared to the existing frequencies in Caucasians and Black people^28^ are presented in Table 4.

**TABLE 4:** Frequency of Rh antigens typed with ID CORE XT among Saudi donors compared to existing reported information from Caucasians and Black people.^28^

Rh antigen (ISBT)	Frequency in this study (%)	Reported in Caucasians (%)	Reported in Blacks (%)
C (RH2)	68.36	68	27
E (RH3)	23.44	29	22
c (RH4)	80.48	80	98
e (RH5)	98.84	98	98
CW (RH8)	0	2	1
V (RH10)	21.75	1	30
hrS (RH19)	97.75	98	99
VS (RH20)	24.84	<0.01	26–40
hrB (RH31)	93.24	98	98

## 6 DISCUSSION

In this study, we report the first molecular analysis of *RHD* and *RHCE* allelic and genotypic variations in the Jazan region of Saudi Arabia. Thus, it serves to enhance our understanding of Rh system variant distributions and the impacts that they have on the safety of transfusions in populations where hemoglobinopathies are prevalent.

*RHD* deletion emerged as the predominant molecular basis for RhD negativity (76.7%) in Saudi donors. This corresponds with earlier studies conducted in both Caucasian and Saudi populations,^26^, ^29^ and in neighbouring countries^30^, ^31^, ^32^; however, it does not correspond with individuals from African backgrounds in which the *RHD* pseudogene is the main cause of the RhD negative phenotype.^33^ This study also revealed genotypes such as non‐weak D types 1, 2, and 3 (18.3%) and weak D type 1 (1.7%) as well as two cases (3.3%) of the *RHDDIIIa‐CE(3–7)‐D* hybrid allele. Despite being common in European populations,^28^, ^29^ weak D types were limited in the present research, reflecting patterns in other non‐European populations.^15^, ^34^ The detection of *RHDDIIIa‐CE(3–7)‐D* hybrid allele in this cohort is a critical finding, as it results in a D‐negative and partial C phenotype, and is linked to *RHCEceVS.03*, which encodes partial c and e antigens and an hrB‐negative phenotype.^35^

A marked *RHCE* allelic diversity among Saudi donors was observed, with a broader spectrum of variants compared to the non‐Saudi cohort. The frequency of *RHCE* alleles in the Saudi population differs from that in other populations.^28^ *RHCE* alleles are very common in African people and mixed‐ethnic populations^36^, ^37^ but are rare in European and Asian populations.^37^ In this report, 708 *RHCE* alleles were analysed in the Saudi donor group, and most donors exhibited RHCE*Ce (44.1%) and RHCE*ce (31.9%). This was followed by RHCE*cE (12.3%), which was moderately frequent, unlike Laget et al. (2022), who reported RHCE*ce as the main allele in the French Guianan population (53.9%).^38^ The variants of the RHCE*ce allele are of great interest in transfusion science because some of these variant alleles encode partial e antigens and may contribute to the incidence of alloimmunisation in transfusion‐dependent patients.^39^ In our population, 9.46% of the Saudis exhibited RHCE*ce*(733G)*, suggesting several observations when compared to common frequency in Africans,^28^ but they are rarely detected in European and Asian populations.^40^ The remaining variants RHCE*ce(733G,1006T), RHCE*ce*AR*, and RHCE*ce(712G) and the hybrid *RHD*r's‐RHCE*ce(733G,1006T) were identified in under 1% of the population, thus rendering them rare in the Saudi donor group. This study found RHCE*ce(733G) and RHCE*ce*AR* at low frequencies, but these alleles are the most frequent variants in sub‐Saharan populations.^41^ On the other hand, RHCE*Ce (48.2%) and RHCE*ce (30.9%) were found to be predominant alleles in non‐Saudi donors (*n* = 220 alleles), with *RHCEcE* at 13.2%. No rare alleles (RHCE*ce*(733G,1006T)*, RHCE*ceAR, RHCE*ce*(712G)*, or RHDr's‐RHCE*ce(*733G,1006T*)) could be found in the non‐Saudi group. Our findings indicate that there is a higher RHCE allele diversity in the Saudi donors, which may be due to ancestry or regional genetic heterogeneity. In addition, approximately 25% of the donors were non‐Saudi and from different ethnicities, despite sharing major *RHCE* allelic patterns.

Allelic diversity within *RHCE* was translated into distinct genotype distributions. The *RHCE* genotype RHCE*ce/RHC*ECe was the most common genotype among the Saudi donors (26.8%), followed by homozygous RHCE*Ce (19.5%) and RHCE*Ce/RHCE*cE (13.3%). Moreover, 12.7% of the donors exhibited RHCE*ce. Interestingly, rare genotypes containing variant alleles (i.e., RHCE*ce/RHCE*ce(*733G*)) were found in 17 donors (4.8%), while homozygous RHCE*ce(733G) (8 Saudi donors) (2.3%) was higher in Saudi donors compared to Caucasians.^27^ Rare genotypes containing variant alleles (i.e., RHCE*ce/RHCE*ce(*733G*)) (4.8%) and homozygous RHCE*ce(733G) (2.3%) could only be detected at lower frequencies, thus rendering them infrequent or rare. This aligns with other studies examining variant *RHCE* alleles (e.g., RHCE*ce(733G)) in populations with African ancestry and hybrid genes.^28^, ^40^ Although frequency patterns were similar between Saudis and non‐Saudis in our population, variant genotypes were less common in non‐Saudis. Only 1.8% of cases exhibited the RHCE*ce/RHCE*ce(733G), while no RHCE*ce(733G)/RHCE*ce(733G) was identified. Importantly, the non‐Saudi group did not exhibit any of the *ceAR, ce(712G)* rare genotypes or hybrid alleles. This suggests that the prevalence of RHCE genotype variants is relatively high, highlighting the need to include RHCE genotyping in blood donor screening, particularly in areas with genetically diverse and prevalent hemoglobinopathies.

The investigated Rh genotypes enabled the identification of Rh variant phenotypes in our donor population. Phenotypic analysis of Rh antigens among Saudi donors showed a near‐universal prevalence of c (RH4), at 80.48%, and e (RH5) at 98.84%, while the C (RH2) antigen was observed in 68.36% of the population and E (RH3) in 23.44% of the population. These results are consistent with our previous serologic data obtained from the Jazan region.^19^ However, the predicted *RHCE* phenotypes, including V, hrS, VS and hrB antigens, have not previously been reported in Saudi Arabia. We reported variant Rh antigens linked to *RHCE* polymorphisms. hrS antigen was highly prevalent (97.75%) and consistent with reported expression in Caucasians and in blacks,^28^, ^40^ hrB was slightly lower (93.24%). Moderately observed expression was identified of the V (21.75%) and VS antigens (24.84%), which are typically present in 30%–40% of people with African ancestry but are uncommon in other populations (<0.01%).^28^, ^40^ This indicates that there were unique *RHCE* variant distributions in the studied population and admixture of Rh allelic variations. Also, these findings highlight the potential clinical significance of these rare phenotypes in the studied population, and the alloimmunisation if positive hrS and hrB antigens are transfused to negative recipients, which then complicates finding compatible blood. Thus, we were able to study the expression of rarely examined Rh antigens using serology. Typically, alloimmunisation is directed against RhCE antigens, as evidenced by previous studies conducted in the Jazan region.^12^, ^13^ The identification of partial antigens highlights the value of *RHCE* molecular typing in both donors and patients to prevent alloimmunisation.

The study is subject to some limitations. The sample size of the non‐Saudi participants may have influenced the detection of rare *RHCE* alleles. However, the study aims to investigate the existing variability of *RHCE* alleles in the Jazan region, where hemoglobinopathies and transfusion‐dependent patients are common. The absence of complete *RHCE* genotyping is another limitation. This study investigated selected single nucleotide variants, which may not capture the full spectrum of *RHCE* variants. Comprehensive *RHCE* genotyping would provide more complete characterisation, as different RHCE*ce(733G) variants can have distinct clinical implications for transfusion safety. The study was conducted in a single region. Thus, further regional and national studies are warranted to understand the diversity of Rh alleles. Limitations in the *RHD* genotyping analysis included the small sample size (*n* = 60), meaning that further large‐scale studies are required to detect new or rare variations. Furthermore, although genotyping targeted significant polymorphisms, complete sequencing was not performed, meaning that some hybrid or recombinant alleles may not have been detected, including those other than weak D types 1, 2, and 3. It is recommended that a national reference database and local transfusion programmes be developed, which can be achieved by extending this molecular study to other areas of Saudi Arabia.

This research presents the first comprehensive molecular characterisation of *RHD* and *RHCE* alleles and genotypes among Saudi blood donors, especially in areas with a high prevalence of hemoglobinopathies, such as the Jazan region. *RHD* deletion was found to be the most significant cause of RhD negativity. RHCE*Ce was the most common allele. RHCE*ce(733G) and other variants, although rare, were also identified, supporting the presence of significant *RHCE* allele diversity. Thoroughly, these findings highlight the risks of alloimmunisation resulting from variant antigens that cannot be detected in standard serology. The accuracy of antigen matching can be enhanced by including molecular genotyping in blood donor screening programmes, especially in genetically diverse areas and locations where hemoglobinopathies are common.

## FUNDING INFORMATION

None.

## CONFLICT OF INTEREST STATEMENT

The authors have no competing interests.

## PATIENT CONSENT STATEMENT

The Institutional Review Board at Jazan Health, Ministry of Health, waived the requirement for informed consent considering the retrospective nature of this study.

## Data Availability

The original contributions presented in the study are included in the article; further inquiries can be directed to the corresponding author.
